# Beneficial microbial signals from alternative feed ingredients: a way to improve sustainability of broiler production?

**DOI:** 10.1111/1751-7915.12794

**Published:** 2017-08-25

**Authors:** Filip Van Immerseel, Venessa Eeckhaut, Robert J. Moore, Mingan Choct, Richard Ducatelle

**Affiliations:** ^1^ Department of Pathology, Bacteriology and Avian Diseases Faculty of Veterinary Medicine Ghent University Merelbeke Belgium; ^2^ School of Science RMIT University Bundoora Vic. Australia; ^3^ School of Environmental and Rural Science University of New England Armidale NSW Australia

## Abstract

More sustainable broiler meat production can be facilitated by the increased use of cheap by‐products and local crops as feed ingredients, while not affecting animal performance and intestinal health, or even improving intestinal health, so that antibiotic usage is further reduced. To achieve this, knowledge of the relationship between the taxonomic and functional microbiota composition and intestinal health is required. In addition, the relationship between the novel feed sources, the substrates present in these feed sources, and the breakdown by enzymes and microbial networks can be crucial, because this can form the basis for development of tailored feed‐type specific solutions for optimal digestion and animal performance.

Chicken meat production is more sustainable and has lower environmental impact than porcine and bovine meat production. Chicken production uses less feed consumed for each kilogram of meat produced and uses less land and water for both farming and feed production (Flachowsky *et al*., 2017). Moreover, the broiler production industry has, for many years, continuously improved animal performance, reflected by ever decreasing feed conversion (kg feed consumed per kg body weight) and reduced time to achieve market weight (Zuidhof *et al*., 2014). This helps reducing the carbon footprint. Drivers for the continuous improvements in performance parameters include genetic selection for high‐performing chicken lines, technological developments in hatching and housing conditions, feed optimization and management practices that support (intestinal) health. Among the latter, the use of antimicrobial growth promoters (antimicrobials added to the diet at low concentrations) is a practice that has been stopped in the EU since 2006, but the use of therapeutic antimicrobials in the animal production industries is still high (Chantziaras *et al*., 2014; Diarra and Malouin, 2014; Van Boeckel *et al*., 2015). Because of concerns about antibiotic resistance and driven by legislation and consumers’ perception and demands, nowadays the use of antimicrobials is decreasing, in some cases leading to an increase in intestinal health problems.

The move away from antimicrobials has led to increasing concerns about gut health that may be compromised by bacterial diseases, enteritis, dysbiosis and poor digestibility and result in poor growth performance of birds. To overcome the increased incidence of gut health problems in animals, many different strategies have been developed. Often feed and drinking water additives are used. These include enzymes (mostly xylanases and glucanases), probiotics, short‐ and medium‐chain fatty acids, herbal compounds, prebiotics and combinations of these, among other molecules (Huyghebaert *et al*., 2011; Caly *et al*., 2015; Gadde *et al*., 2017). In addition, research on the modes of action of these products and on underlying microbiota‐ or host‐related functions involved in intestinal health has led to a better understanding of the intestinal ecosystem in health and disease (Stanley *et al*., 2014).

In production animals, a key issue is digestibility of nutrients and energy harvest from the diet. Although brush border enzymes and exogenous enzymes (in diet) are important in this regard, the gut microbiota has a key function in carbohydrate, fat and protein digestion as well (Flint *et al*., 2012; Scott *et al*., 2013). In case of incomplete digestion of nutrients in the small intestine, the excess nutrients from an energy‐rich diet can fuel small intestinal bacterial overgrowth, potentially resulting in inflammation and in some case even severe disease (e.g. necrotic enteritis by *Clostridium perfringens*) (Shojadoost *et al*., 2012; Kiarie *et al*., 2013; Pan and Yu, 2014; Wu *et al*., 2014). To avoid such problems, different feed additives have been used to support host digestive processes. Exogenously added enzymes play a role and have been administered for many years as feed additives to enhance digestion (Amerah *et al*., 2017; Gonzalez‐Ortiz *et al*., 2017).

Different feed ingredients are used in poultry diets in different regions around the world, with wheat and corn the most predominant cereals, and soya the most important protein source. Substituting (partly) these high‐quality feed ingredients with alternative feed materials, often by‐products from other industries or local products, could be a valuable way to further enhance sustainability of poultry production worldwide. Such alternative feed sources are often cheaper but may be less digestible, so animal performance can be reduced and the incidence of gut health problems can increase. Examples of alternative feed sources include distiller's dried grains with solubles (DDGS), sunflower cake meal, cassava pulp, palm kernel cake/meal, lupines, sweet potatoes, insects, worms, seaweed and many others, often depending on local availabilities (Ravindran, 2013). Improving the digestibility of poorly digestible ingredients is a valuable objective where microbial biotechnological solutions can fill a need. Complex indigestible substrates, such as plant cell wall polysaccharides, require several enzymatic reactions in cascade in order to achieve full catabolism. The microbiota can break down these complex molecules, to an extent, and thus harvest energy that can, at least partly, be used by the host. In this process, microbial networks are essential, as different steps in complex substrate breakdown involve different bacterial taxa, with their specific enzymes, each participating in a certain step or process depending on the type of substrate present (Flint *et al*., 2012, 2015; White *et al*., 2014). Not only breakdown of substrates but also the end metabolic products that are produced during fermentation are of utmost importance, as these metabolites, for example short‐chain fatty acids, steer intestinal health (Russell *et al*., 2013; Zhang and Davies, 2016). Microbial biotechnology can be of use in different disciplines that can contribute to more sustainable poultry (and other animal species) production in the future:

1. Defining the microbiota composition and microbial functional pathways in the chicken's intestinal tract and their relation with intestinal health and performance.

Insight in microbial community organization and characterization of the functional activities of these communities is a key topic nowadays and essential to understand how microbiota–host interactions drive intestinal health and function. While some work has been done on describing microbiota composition in the different segments of the intestinal tract of chickens (Oakley *et al*., 2014; Antonissen *et al*., 2016; Stanley *et al*., 2016), there is a clear need to further investigate microbial taxa that are over‐ or underrepresented in the gut of chickens in specific experimentally induced or field cases of intestinal health problems. Even then, it will be of importance to evaluate the significance and possible causal relationships of these findings, as data derived from 16S rRNA sequencing are descriptive and do not necessarily reflect functionally and pathologically relevant changes. As an example, isolating and evaluating the behaviour of specific species in intestinal health models or studying their behaviour *in vitro* (e.g. substrate preference, metabolite production) can be valuable (De Maesschalck *et al*., 2015; Eeckhaut *et al*., 2016). Even more, future studies should target pathways present in the microbiota, as can be done using metagenomic approaches. This can add information on the functionality of a microbiota that is linked with intestinal health, and ideally, with performance.

A particular challenge in determining the contribution of gut microbiota to poultry productivity is understanding the metabolic potential and role of species within the microbiota that have not been cultured and characterized. 16S rRNA sequencing has shown that there are typically many members of the broiler gut microbiota that are only distantly related to strains that have currently been characterized. There are new culturing methods now available which may capture some of these unknown strains (Browne *et al*., 2016) and there are also advanced metagenomic sequence assembly methods that have been successfully used to construct whole genomes of novel organisms within the microbiota (Sangwan *et al*., 2016). There are great opportunities to apply these advanced microbiological methods to the chicken microbiota in order to improve our understanding of how the microbiota may influence the utilization of novel feed inputs.

- Defining metabolic profiles in the gut and their relation with intestinal health.

While 16S rRNA sequencing and metagenomics can yield information on abundances of taxa and abundances of genes encoding functional activities, including DNA biomarker estimation (Segata *et al*., 2011), information on gene expression but especially on microbial metabolic profiles in the intestine will be essential to understand microbiota–host interplay in the gut. Products from polysaccharide fermentation (mainly lactate, short‐chain fatty acids and gases) have been well studied in relation to intestinal health (Havenaar, 2011; Russell *et al*., 2013; Onrust *et al*., 2015). This is not the case for products from protein and fat degradation, although beneficial effects of, for example, indole, which is formed during degradation of tryptophan, are well described. More in‐depth studies on the relationship between feed sources, their constituents and the microbial taxa that degrade these substrates, the microbial end‐products and their dependence on the microbiota composition itself, are key targets for future research. This will enable moves towards optimal intestinal health using nutritional approaches. Ideally, besides the production of enzymes or other actions, probiotics could be designed to produce a set of microbial signals that reduce inflammation, inhibit pathogen overgrowth and favour digestion when delivered in‐feed together with its substrates. The use of a complex of probiotic strains, which operate as a network to feed each other or produce different agonistic end metabolites, should be considered. Butyrate production is the most well‐known example of a beneficial end‐product (Onrust *et al*., 2015).

- Identifying enzymes and enzymatic degradation pathways needed to break down novel feed sources and application of tailored solutions for specific feed types

Enzymes have been used as feed additives in poultry diets for many years. Their main target so far was to reduce viscosity and decrease the levels of the so‐called antinutritional compounds (Kiarie *et al*., 2013). A lot of these viscous compounds are soluble non‐starch polysaccharides (NSPs, e.g. pectins, arabinoxylans and beta‐glucans), typically present at high concentrations in specific feed ingredients (e.g. rye, barley and wheat; Knudsen, 2014). These NSPs are very complex because they can differ in molecular weight, side‐chain length and composition, linkage types between molecules, and many more (Knudsen, 2014). The substrates are very heterogeneous, even within the same type of compounds (e.g. arabinoxylans), and thus, the enzymes needed to be tailored for these substrates in order to degrade them. Here, the challenge for microbial biotechnology is evident: first, there is a definite need to characterize, in detail, the composition of feed ingredients that can potentially replace high‐quality cereals and soya; second, there is a strong need to identify enzymes (or mixes of them) that are capable of degrading these specific substrates. In this way, a feed‐specific enzyme mixture could be designed and supplemented when specific sustainable feed ingredients are used to, at least partly, replace conventional cereals and protein sources in feed. Furthermore, there is an opportunity to develop microbial mixtures that form a substrate‐degradation product of end metabolites beneficial for intestinal health. Thus, mass‐scale stable production of these mixtures is a biotechnological challenge. All these above‐mentioned ideas need coordinated approaches that combine classical microbiology, analytical chemistry, but also high‐throughput sequencing and bioinformatics.

Summarizing, more sustainable broiler meat production can be facilitated by the increased use of cheap by‐products and local crops as feed ingredients, while not affecting animal performance and intestinal health, or even improving intestinal health, so that antibiotic usage is further reduced. Therefore, knowledge of the relationship between the taxonomic and functional microbiota composition and intestinal health is required, and the relationship between the feed sources, the substrates present, and the breakdown by enzymes and microbial networks can be crucial, because this can form the basis for development of tailored feed‐type specific solutions for optimal digestion and animal performance.

## Conflict of interest

The authors declare that there is no conflict of interest.
